# Impact of Interventions on Peri-Intubation Hypoxemia and Hypotension in Critically Ill Patients: Systematic Review and Meta-Analysis

**DOI:** 10.5811/westjem.41210

**Published:** 2025-09-27

**Authors:** Christine E. Ren, Jessica V. Downing, Stephanie Cardona, Isha Yardi, Manahel Zahid, Kaitlyn Tang, Vera Bzhilyanskaya, Priya Patel, Ali Pourmand, Quincy K. Tran

**Affiliations:** * Oregon Health and Science University, Department of Emergency Medicine and Critical Care Medicine, Portland, Oregon; † R. Adams Cowley Shock Trauma Center, Program in Trauma, Baltimore, Maryland; ‡ University of Maryland School of Medicine, Department of Emergency Medicine, Research Associate Program, Baltimore, Maryland; § The Mount Sinai Hospital, Department of Critical Care, New York, New York; || George Washington University School of Medicine and Health Sciences, Department of Emergency Medicine, Washington, DC; ** University of Maryland School of Medicine, Baltimore, Maryland

## Abstract

**Introduction:**

Emergent endotracheal intubation is common in critically ill patients. Underlying pathophysiologic derangements puts these patients at increased risk of peri-intubation major adverse events (MAE) and have been associated with higher morbidity and mortality. Investigating the impact of interventions in the peri-intubation period on the rate of peri-intubation hypoxemia and hypotension can help improve management of emergent airways.

**Methods:**

We searched PubMed, Embase, and Scopus databases from their beginning through April 2024 to identify randomized controlled trials (RCT) evaluating interventions to prevent peri-intubation hypoxemia and hypotension. Random-effects meta-analysis was used for the outcomes of peri-intubation hypoxemia and hypotension. We used the Cochrane risk-of-bias tool and Cochrane Q-statistic and I2 to assess the quality and heterogeneity of the included studies, respectively.

**Results:**

We included 16 RCTs included in our analysis with a total of 7,778 patients. All studies reported incidences of peri-intubation hypoxemia, and 11 studies reported rates of hypotension. One study had some concern of bias; otherwise all others were found to have low risk of bias. The examined interventions were associated with a 25% reduction in rates of hypoxemia (OR 0.748, 95% CI 0.566 – 0.988, P = .04). The subgroup of preoxygenation techniques showed a 63% reduction in rates of hypoxemia (OR 0.37, 95% CI 0.23 – 0.61, P < .001). Interventions to prevent hypotension were not associated with a significant decrease in rates of peri-intubation hypotension (OR 0.848, CI 0.676 – 1.063, P = .15).

**Conclusion:**

Preoxygenation interventions, in the form of noninvasive ventilation, are associated with lower odds of hypoxemia in the peri-intubation period. More research is needed to determine whether interventions can be successful at preventing cardiovascular collapse.

## INTRODUCTION

Peri-intubation major adverse events (MAE), including hypoxemia, hypotension, and cardiac arrest, occur in up to 30% of patients intubated in the emergency department (ED), intensive care unit (ICU), or on the medical floors. Among these patients, emergency airway management often occurs in the setting of physiological derangements, such as hypoxemia, acidosis, and hypotension, that increase risk for peri-intubation MAEs; as a result, almost 1 in 3 patients intubated emergently in the ED or ICU will experience a peri-intubation MAE, such as hypoxemia (15%) or cardiovascular collapse (18%).^1^ Studies have consistently demonstrated that peri-intubation hypotension and hypoxemia are strongly associated with increased in-hospital mortality, underscoring the importance of optimizing the airway management process.^2^–^5^ Pre-intubation hypotension has been identified as a risk factor for subsequent post-intubation cardiac arrest and prolonged ICU stays.^6^,^7^

Several studies have examined interventions aimed at reducing patient risk during the peri-intubation period. These include preoxygenation techniques (such as use of high flow nasal cannula [HFNC] or various forms of positive pressure ventilation [PPV] to increase mean airway pressure and alveolar recruitment); implementation of airway adjuncts (such as a gum elastic bougie or video laryngoscopy [VL]) to reduce apneic time and improve first-pass success; and preemptive fluid boluses during induction to increase venous return.^5^–^8^ The Society of Critical Care Medicine recently devised practice guidelines for rapid sequence intubation (RSI) including suggestions for preoxygenation with HFNC or non-invasive ventilation (NIV) in high-risk patients.^9^ However, gaps in high-quality evidence regarding optimal emergency airway management still remain, resulting in ongoing controversies in airway approaches and a wide range of clinical practices.

In this systematic review and meta-analysis, we examine the association between these interventions and rates of peri-intubation MAEs, including hypoxemia, hypotension, and cardiac arrest. In this study we aimed to contribute to the understanding of effective strategies in emergency airway management and guide clinical decision-making.

## METHODS

### Search and Selection Criteria

Our study was conducted in accordance with the 2020 Preferred Reporting Items for Systematic Reviews and Meta-Analyses (PRISMA) statement, and we used the PRISMA checklist when writing our report.^10^ We searched PubMed, Embase, and Scopus on June 1, 2021, September 4, 2022, and April 23, 2024, to identify eligible studies. The search terms used were ((adverse) AND (events) OR (hypoxemia) OR (hypoxia) OR (desaturation) OR (hypotension) OR (cardiac AND arrest) OR (hemodynamic OR cardiovascular) AND (collapse)) AND ((endotracheal AND intubation) OR (emergency AND airway AND management) OR (peri-intubation)). We also searched included articles to identify additional potentially eligible articles. We did not contact authors for more data.

**Population Health Research Capsule**

What do we already know about this issue?

*Hypoxemia and hypotension are common during intubation. While interventions have been studied, evidence gaps remain as to their efficacy*.

What was the research question?

*What is the association between interventions and rates of peri-intubation hypoxemia and hypotension?*

What was the major finding of the study?

*Noninvasive ventilation or high flow oxygen preoxygenation decreased rates of hypoxemia (OR 0.37, 95% CI 0.23 – 0.61, P < .001)*.

How does this improve population health?

*Preoxygenation can reduce peri-intubation hypoxemia. Data on interventions to reduce peri-intubation hypotension are limited*.

Any randomized controlled trial (RCT) investigating the effects of interventions on peri-intubation hypoxemia, hypotension, or cardiac arrest were eligible for inclusion. We excluded the following studies: 1) did not have specified criteria for adverse events; 2) included pediatric patients only or did not differentiate outcomes among adult and pediatric patients; 3) included operating room, post-anesthesia care unit, or out-of-hospital (ie, emergency medical services) intubations; 4) were published in non-English language; 5) were systematic reviews, meta-analyses, observational studies, conference publications, case reports, or abstracts; and (6) did not have primary or secondary outcomes of hypoxemia, hypotension, or cardiac arrest. We screened enrollment databases, locations, and years for each study to identify potential duplicate reporting of data across studies. We elected to omit observational studies, given a previous meta-analysis of literature that showed numerous RCTs on peri-intubation adverse events,^1^ which thus allowed for adequate power and sample size while minimizing risk of bias, lowering the heterogeneity of the effect size, and strengthening the level of evidence of findings.

Our systematic review and meta-analysis was registered with PROSPERO (CRD42022342134). We used Covidence (www.covidence.org, first accessed on November 25, 2022) to review eligible studies. Each study title, abstract, and full text was reviewed independently by two investigators; disagreements were resolved by a third senior investigator. This systematic review, conducted solely through the examination of secondary data and without any direct interaction with human subjects or primary data collection, was not subject to formal ethics approval.

### Outcomes

We examined three separate MAEs individually, as the interventions recommended to prevent or address them are distinct. We examined the rates of hypoxemia, hypotension (both as defined by study authors), and cardiac arrest in the peri-intubation period. The peri-intubation period was also defined by study authors.

### Quality Assessment

We assessed the quality of included RCTs using the Cochrane risk-of-bias (RoB) 2 tool, which evaluates risk of bias in randomization, deviations from study protocol, outcome measurement, selection of reporting, and effect of missing data.^11^ Based on the investigators’ assessment of each domain, the tool characterized risk of bias as “low,” “some concerns,” or “high.” If any single domain is determined to have “high” risk or “some concern” for bias, the entire study is judged to have this level of bias. Two independent investigators assessed the quality of each study, and any disagreement was adjudicated by a third investigator. Heterogeneity was assessed by the Cochrane Q statistic, which tests against the null hypothesis that all analyzed studies share a common effect size and I^2^ statistic, which determines the percentage of total variance expected to occur because of differences in effect size across included studies.

### Data Collection

Data was extracted into a standardized Microsoft Excel spreadsheet (Microsoft Corporation, Redmond, WA). We included each study’s year of publication, geographic location, dates of data collection, setting (ED, ICU, or mixed), intervention studied, study outcomes of interest, and patients’ clinical variables (if provided), such as demographics, vital signs, laboratory values, pre-intubation intravenous fluid (IVF) or vasopressor use and preoxygenation techniques, reason for intubation, and presence of MAEs. Interventions were subsequently categorized as IVF bolus administration, preoxygenation with NIV, preoxygenation with HFNC, or other. Data was extracted independently by two investigators and then compared for discrepancies; any discrepancies were discussed and resolved by the investigators. All data was adjudicated by group consensus before finalization.

### Statistical analysis

We presented continuous data as mean (SD); all data originally presented as median (interquartile range [IQR]) were converted into mean (SD) using VassarStats (http://vassarstats.net/median_range.html, last accessed October 26th, 2024).^12^ Categorical data were presented as numbers and percentages.

We used random-effects models to measure the prevalence of MAEs across the pooled population in our studies. Any two studies reporting the same results were eligible for analysis. Results were reported as odds ratios (OR), with a 95% confidence interval (95% CI) for each outcome. Moderator analyses were performed to identify potential sources of heterogeneity and to compare subgroups, based on studies’ World Health Organization (WHO) region, clinical setting (ICU, ED, ward or mixed), type of adverse event, and type of intervention (IVF bolus administration, preoxygenation with NIV, preoxygenation with HFNC, or other). We studied the effect of composite interventions on primary outcomes of hypoxemia and hypotension. Similar intervention groups were then categorized together—preoxygenation techniques, intubation tools and techniques, induction agents, and IVF administration. These categories were determined based on the selected studies and were not determined a priori. We performed meta-analyses of separate types of interventions if there were adequate studies in each category of interventions. If studies in the categories were too small or varied in intervention approach, we provided a narrative review of our findings.

Sensitivity analysis was performed by “remove-one-study” random-effects meta-analysis, which sequentially removes one study at a time to assess the impact of each individual study on the overall effect size. We assessed publication bias using the Begg and Egger tests (with *P* > .05 suggesting low likelihood of publication bias for each of these tests), the Orwin fail-safe N test (which predicts the number of missing or future studies that might change the effect size of our primary outcome), and the funnel plot. We performed all statistical analyses using the software Comprehensive Meta-Analysis (www.meta-analysis.com).

## RESULTS

### Study Selection and Summary

**Figure 1:**
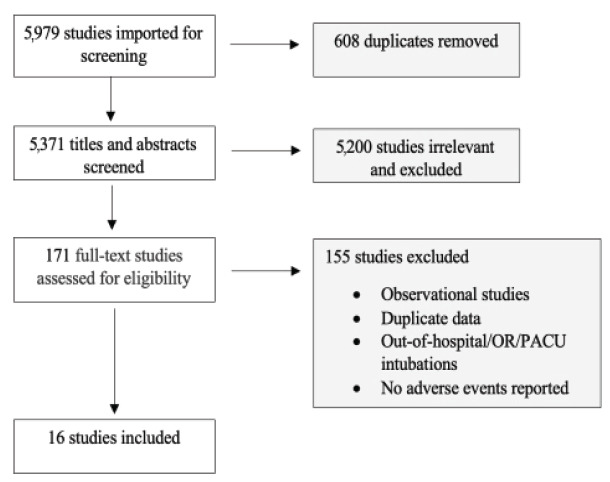
Flow diagram for the study selection process. Adapted from the PRISMA 2020 statement. *OR*, operating room; *PACU*, post-anesthesia care unit; *PRISMA*, Preferred Reporting Items for Systematic reviews and Meta-Analyses.

We included 16 RCTs (Figure 1) with a cumulative 7,778 patients (range 49–1417; Supplemental Table 1). We considered duplicate enrollment likely in two pairs of studies: Casey et al 2019^13^ and Janz et al 2019,^14^ and Semler et al 2017^15^ and Janz et al 2018.^16^ In each of these cases, the study pairs in question investigated distinct interventions; thus, we included all four studies in our analysis (Supplemental Table 1). All but one study^17^ included intubations in the ICU; five studies^14^,^17^–^20^ included intubations that occurred in the ED. Hypoxemia was the primary outcome of interest for eight^13^,^15^,^16^,^18^,^18^,^21^–^23^ and was reported in all included studies; in studies reporting hypoxemia as a primary outcome, hypoxemia was defined as SpO_2_ <80% in four studies,^13^,^22^–^24^ <85% in one study,^18^ and <90% in one study^15^; two studies^16^,^21^ examined the lowest saturation without defining a cutoff.

Hypotension was the primary outcome of interest for four^14^,^16^,^25^,^26^ studies and was reported by an additional seven.^13^,^18^,^20^,^23^,^24^,^27^,^28^ In studies that reported hypotension as a primary outcome, hypotension was defined as a systolic blood pressure (SBP) <65 in two studies,^14^,^26^ a change in mean arterial pressure (MAP) from baseline in one study,^25^ and the lowest SBP without a predefined range in one study.^16^ First-pass success was the primary outcome for five studies.^17^,^19^,^20^,^27^,^28^ Interventions included pre-oxygenation techniques (including NIV,^18^,^21^,^23^,^24^ HFNC,^22^,^23^ bag-valve mask [BVM]^13^), intubation tools and techniques (use of video laryngoscopy VL,^20^,^27^,^28^ use of gum-elastic bougie,^17^,^19^ patient positioning [ramped or sniffing],^15^ and the use of a pre-intubation checklist^16^), induction agent (reduced dose etomidate or a combination of ketamine and propofol^25^) and IVF administration (500 millilitersmL during induction^14^,^26^).

### Study Quality

**Figure 2:**
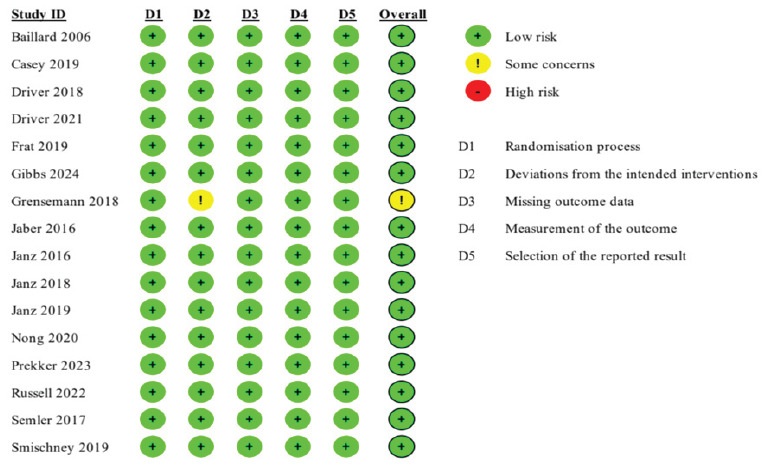
Cochrane risk-of-bias^11^ assessment for included studies. Each rating is based on consensus between two investigators.

Fifteen of the 16 included studies were judged to have a low risk of bias (Supplemental Table 1). Grensemann 2018^27^ was deemed to have some concern of bias, given that their study did not describe whether there was analysis to estimate effect of assignment to an intervention, such as intention to treat or per protocol analysis (Figure 2).

### Outcome 1: Hypoxemia

Hypoxemia was reported in 16 studies and was the primary outcome for eight studies. The interventions examined were associated with an estimated 25% reduction in rates of hypoxemia (OR 0.748, 95% CI 0.566 – 0.988, *P* = . 04; Figure 3a). The prediction interval was wide and crossed 1 (0.280–1.997), indicating that the interventions studied could be expected to correlate with anywhere from a 70% reduction of hypoxemia to a 99% increase in rates of hypoxemia. There was a high degree of heterogeneity. The *P*-value for the Q statistic was 0.001, which suggests that the true effect varied among the included studies. The I^2^ statistic was 74%.

Moderator analysis suggested that among the interventions used to prevent hypoxemia, only studies using NIV (OR 0.35, 95% CI 0.17 – 0.72, *P* < .01) or other forms of preoxygenation were associated with a significant reduction in rates of hypoxemia (OR 0.39, 95% CI 0.23 – 0.67, *P* = .001) (Table 1). Multivariate meta-regression suggested that higher body mass index (BMI) was associated with higher rates of hypoxemia (Pearson correlation coefficient [r] 0.35, 95% CI 0.09 – 0.61, *P*=.01), and that patients intubated for respiratory failure had lower rates of peri-intubation hypoxemia than those with other indications for intubation (r −4.9, 95% CI −9.1 − [− 0.77], *P* = .02) Table 2).

Sensitivity meta-analysis using a “one-study-removed” approach identified similar results to our original analysis, suggesting no single study disproportionately affected our findings (Figure 3b). The Begg and Egger tests suggested low likelihood of publication bias (*P* = .39 and *P* = .36, respectively). The Orwin fail-safe determined that eight future or missing studies with an effect size of 1.6 favoring control would be needed to negate the significance of our findings. Our funnel plot demonstrated relative symmetry, also supporting a low likelihood of publication bias (Figure 3c).

We then examined the effects on hypoxemia of studies that looked at preoxygenation techniques, which included NIV,^18^,^21^,^23^,^24^ HFNC,^22^,^23^ and BVM.^13^ These were associated with a 63% reduction in rates of hypoxemia (OR 0.37, 95% CI 0.23 – 0.61, *P* < .001; Figure 3d). The prediction interval was large and crossed 1 (0.09 – 1.55), suggesting that the studies that looked at preoxygenation techniques could range from a 90% reduction of hypoxemia to a 55% increase in hypoxemia. There was substantial heterogeneity with an I² statistic of 62%. The *P*-value for the Q statistic was .02. Sensitivity meta-analysis using “one-study-removed” suggested no single study disproportionately affected our findings (Figure 3e). The funnel plot of preoxygenation techniques on hypoxemia exhibited more studies laying to the left of midline, suggesting presence of publication bias and that most of these studies reported positive results from the intervention (Figure 3f).

Moderator analyses showed that studies from the Americas region^13^,^18^ were associated with low heterogeneity when compared to the European studies^21^,^22^,^23^ (Table 3). Multivariate meta-regression did not reveal any clinical factors that were associated with odds of hypoxemia (Table 4). Evaluation of the effect of airway adjuncts, such as gum-elastic bougie,^17^,^19^ showed no significant changes in rates of hypoxemia.

### Outcome 2: Hypotension

Eleven studies reported rates of hypotension, which was the primary outcome for three. The interventions examined were not associated with a significant decrease in rates of hypotension (OR 0.848, CI 0.676 – 1.063, p = 0.15; Figure 4a). The I statistic was 0, because no studies reported a significant change in rates of hypotension associated with the intervention.

Sensitivity meta-analysis using “one-study-removed” suggested no single study disproportionately affected our findings (Figure 4b). Begg and Egger’s tests suggested low likelihood of publication bias (p = 0.52 and p = 0.28, respectively). Our funnel plot demonstrated symmetry, suggesting a low likelihood of bias, but does raise the possibility of a small study effect with more studies associated with a higher standard error (Figure 4c).

Studies that looked at fluid boluses^14^,^26^ as interventions, showed no significant change in rates of hypotension.

### Outcome 3: Cardiac Arrest

Eleven studies reported cardiac arrest as a secondary outcome; their interventions were not associated with a reduction in the rates of cardiac arrest (OR 0.962, 95% CI 0.6011–1.539; *P* = .87; Figure 5a). The overall reported prevalence of peri-intubation cardiac arrest was very low (the overall pooled prevalence was <1.3%, 1.2% among intervention groups as 1.3% among control groups). Sensitivity meta-analysis using a “one-study-removed” approach identified similar results to our original analysis (Figure 5b). There were not enough studies to perform moderator analyses.

## DISCUSSION

We found that previously investigated interventions to minimize peri-intubation hypoxemia among patients undergoing emergent intubation in the ED or ICU demonstrated success, while those intended to minimize peri-intubation hypotension did not. Specifically, studies investigating NIV or other forms of preoxygenation were associated with a decreased risk of peri-intubation hypoxemia. The administration of IVF boluses in the peri-intubation period was not associated with decreased rates of hypotension. No examined interventions impacted rates of cardiac arrest.

The heterogeneity in our study was high, likely due to diversity in the clinical settings and geographic locations of included studies (expected to reflect different practice patterns and resource use). Despite this, our sensitivity analysis confirms that our findings were not overly influenced by any single study, suggesting that our findings are likely accurate, while not precise.

Most of the studies included here investigated the effect of NIV—via BVM-assisted breaths with a positive end-expiratory pressure (PEEP) valve, bi-level positive airway pressure (BiPAP) ventilation with a face mask and mechanical ventilator, or a combination of techniques—on peri-intubation hypoxemia. Three studies compared the use of NIV to low-flow preoxygenation techniques such as nasal cannula (NC), bag-mask device without manual ventilation, or non-rebreather mask (NRB); higher rates of severe hypoxemia (SpO_2_ < 80%) were found in the low-flow oxygen groups.^13^,^18^,^21^ One compared NIV to HFNC, which can deliver oxygen flow rates of up to 60 liters per minute (LPM) (for comparison, NRB masks typically deliver around 15 LPM) and a fraction of inspired oxygen (FiO_2_) as high as 100%.^23^ While we did not identify any significant difference in overall rates of severe hypoxemia (SpO_2_ < 80%), we did find that, among patients with moderate to severe pre-intubation hypoxemia (defined as PaO_2_:FiO_2_ ≤ 200), preoxygenation with NIV was associated with lower rates of severe hypoxemia during the intubation.

The means through which NIV were applied across studies varied widely. Gibbs et al compared preoxygenation with NIV using pressure support of >10 centimeters water (cm H_2_O) and a PEEP of >5 cmH_2_O with FiO_2_ 100% to either a NRB or a bag-mask device without manual ventilation.^18^ Both trial groups underwent preoxygenation with these methods for three minutes before induction, and intubators were permitted to use a BVM between anesthesia induction and initiation of laryngoscopy. The NIV group was found to have lower rates of hypoxemia, with a greater effect noted in patients with a higher BMI. Nong et al compared BVM with a PEEP of 5 cmH_2_O and BiPAP with inspiratory pressures of 12 – 20 cmH_2_O and PEEP of 5 cmH_2_O, with the BiPAP group continuing NIV throughout the intubation procedure.^24^ Patients allocated to the BVM were ventilated for three minutes prior to fiberoptic intubation accompanied by passive oxygenation via NC.

Patients in the BiPAP group underwent fiberoptic intubation through a sealed hole in the BiPAP face mask, allowing continuation of ventilation during the intubation procedure. While this study technically examined preoxygenation via NIV, it did not examine a type of preoxygenation easily implemented in most centers. In the BiPAP group, SpO_2_ levels before and during intubation were higher and rates of severe hypoxemia were lower, suggesting a positive impact of this novel approach. Other non-standard approaches described may be more feasible: Jaber et al compared NIV using pressure support of 10 cmH_2_O and PEEP of 5 cmH_2_O with FiO_2_ of 100 % to NIV plus HFNC with settings of flow 60 LPM and FiO_2_ 100%; the combination of techniques was better at preventing peri-intubation hypoxemia.^22^ Overall, NIV appears effective in reducing risk of peri-intubation hypoxemia compared to other preoxygenation techniques. While therapies such as NC and NRB provide passive oxygenation, NIV also unloads respiratory muscles, lowering work of breathing and diaphragmatic work, increasing tidal volume and ventilation, and providing high fraction of inspired oxygen.^29^ In doing this, patients have a higher respiratory reserve (higher pO_2_ and lower pCO_2_ levels) when induction and paralytic agents are administered, leading to lower rates of hypoxemia.

The use of NIV is not without risk; reported complications include gastric insufflation, aspiration, barotrauma, and agitation.^30^ Three studies included in our meta-analysis compared rates of witnessed aspiration or a new infiltrate on post-intubation chest radiograph (CXR) between patients receiving NIV and those receiving low-flow oxygen therapy, and did not find any difference between the two groups.^13^,^18^,^21^ Similarly, there was no difference regarding rates of regurgitation, gastric distention, CXR infiltrates, hypotension, or agitation in patients who received NIV compared to HFNC or HFNC plus NIV.^22^,^23^ Although NIV exposes patients to positive pressure ventilation, which may not be tolerated by patients with hemodynamic compromise and may progress to cardiovascular collapse, this change in physiology mimics that introduced by invasive mechanical ventilation, and as such does not introduce a new risk profile. The studies included here that investigated rates of hypotension between patients preoxygenated with NIV or high and low-flow oxygen did not identify any significant differences in rates of hypoxemia.^23^,^24^

Studies examining techniques to improve first-pass success did not demonstrate any significant difference in rates of hypoxemia or hypotension, although they were not powered to do so. Driver et al’s 2021 comparison of gum-elastic bougie and traditional stylet demonstrated a 2.2% increase in absolute risk of hypoxemia in the bougie group.^17^ Prekker et al found that VL was associated with 0.7% lower absolute risk of hypoxemia and 1.3% absolute risk of hypotension when compared to direct laryngoscopy, with no improvement in first-pass success despite improvement in glottic visualization.^20^ Grensemann et al, who studied the use of an endotracheal tube-mounted camera, found no difference in first-pass success, hypoxemia, or hypotension between these two groups,^27^

Of interest, in a meta-regression of studies reporting use of HFNC and percentage of patients intubated for respiratory failure, those intubated for respiratory failure had lower odds of peri-intubation hypoxemia. Use of HFNC did not impact rates of peri-intubation hypoxemia in this analysis. Based on these findings and clinical experience, we speculate this may be reflective of variations in pre-intubation resuscitation strategies: specifically, that clinicians are more likely to prioritize aggressive preoxygenation specifically (perhaps with NIV) among patients with respiratory failure, leading to lower rates of peri-intubation hypoxemia. Our data give limited insight into the explanation for this surprising finding but highlights it as an area for further investigation.

Two RCTs investigating the effect of a peri-intubation IVF bolus on rates of cardiovascular collapse were included in our meta-analysis; neither showed a difference in outcomes.^14^,^26^ Both studies included a set or average bolus volume of 500mL; it is unclear whether a different amount would have generated a different outcome. None investigated preemptive use of vasoactive medications. Other studies have shown promising results in the prevention of peri-intubation cardiovascular collapse, although the means through which these results were accomplished are difficult to narrow down. Jaber et al investigated the implementation of an “ICU intubation bundle,” which included 10 components: preoxygenation with NIV; presence of two operators; RSI; cricoid pressure, capnography, protective ventilation, fluid loading, and preparation and early administration of sedation; and vasopressor use if needed. The authors found significantly lower rates of life-threatening complications in the intervention group (21% vs. 34%, *P* = .03), and a 50% reduction in the incidence of severe hypoxemia and severe cardiovascular collapse.^31^ Further research is needed to determine which specific interventions, (such as the use of specific induction agents or early administration of vasopressors), can reduce rates of peri-intubation cardiovascular collapse.

## LIMITATIONS

Most included patients were intubated in an ICU; so our findings may not be generalizable to other settings or populations. The implementation of interventions varied widely between studies. The number of included studies was small, particularly given the heterogeneity in practice location, practice setting, and interventions. We were unable to provide a standardized range for hypoxemia or hypotension given the inconsistent definitions across our included studies. This introduced significant heterogeneity to our results and highlights the need for consensus of definition for peri-intubation adverse events.

There were differences overall in the control groups of the various studies we included. Some compared intervention to intervention in their studies, and some included overlapping interventions. Studies that looked at airway adjuncts, VL, endotracheal tube-mounted cameras, induction medications, and checklists all employed various modalities of preoxygenation in both their control and intervention groups. Although this closely mirrors the heterogeneity of approach in clinical practice, it may result in multiple confounding factors.

Only three studies investigated hypotension as a primary outcome, and both only examined the role of IVF boluses; additional research is needed to evaluate the impact of other interventions. The overall number of cardiac arrests observed in these studies was low, and none were powered to identify a change in the incidence of cardiac arrest. Lastly, multiple studies reported similar clinical outcomes as separate MAEs (eg, addition of vasopressors and hypotension, hypotension and cardiac arrest, or hypoxemia and cardiac arrest). It is likely that separately reported MAEs could have been related or overlapped.

## CONCLUSION

Pre-oxygenation with NIV was associated with lower odds of peri-intubation hypoxemia. More trials are needed to determine whether interventions can successfully prevent peri-intubation hypotension.

## Supplementary Information



## Figures and Tables

**Figure 3a:**
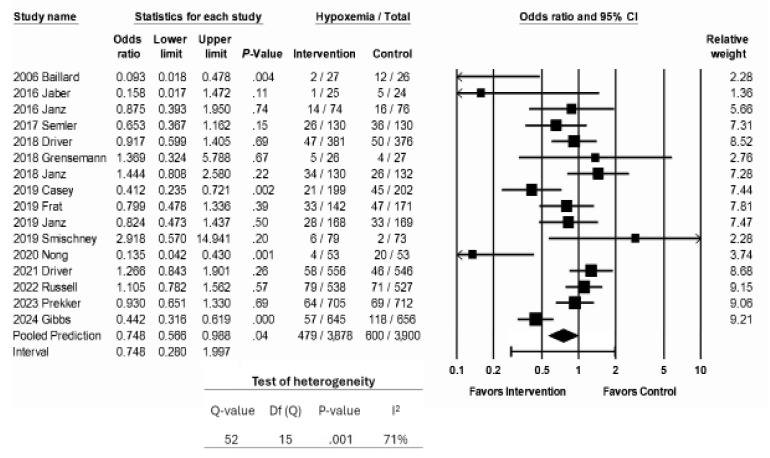
Association of interventions with rates of peri-intubation hypoxemia.

**Figure 3b:**
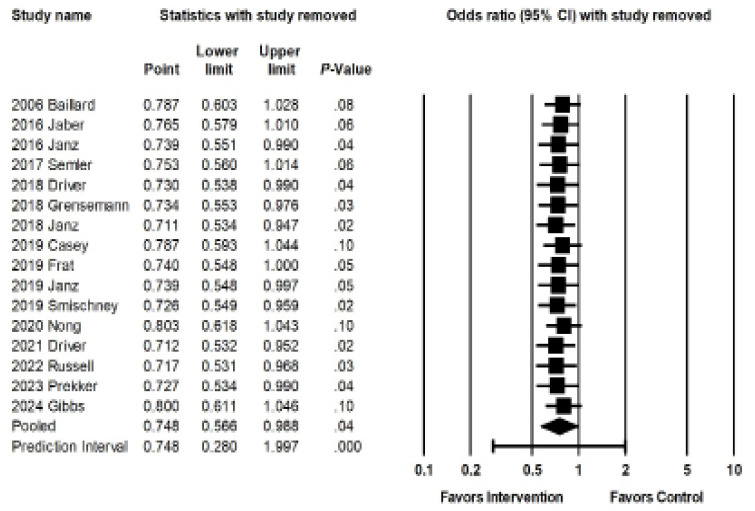
Sensitivity analysis for the association of interventions with rates of peri-intubation hypoxemia.

**Figure 3c:**
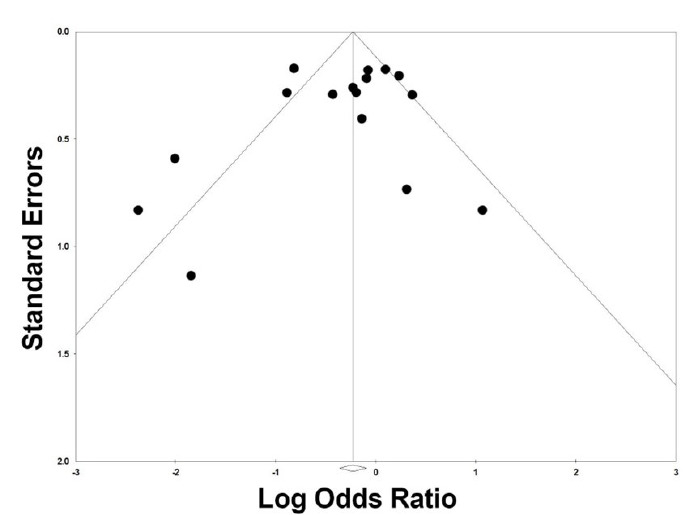
Publication bias funnel plot of studies investigating effect of interventions on hypoxemia.

**Figure 3d:**
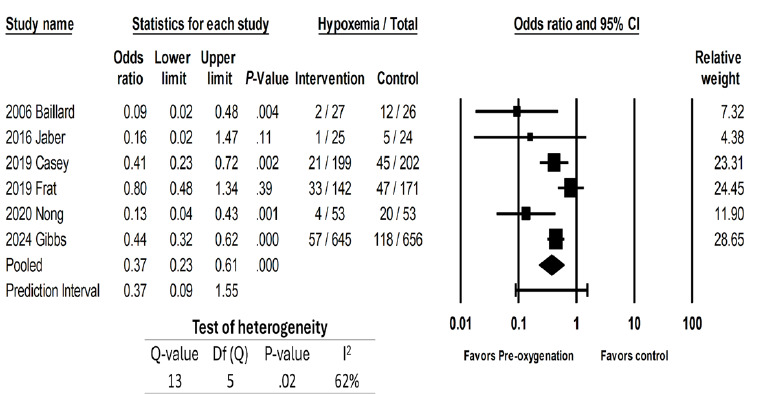
Association of preoxygenation techniques with rates of peri-intubation hypoxemia.

**Figure 3e:**
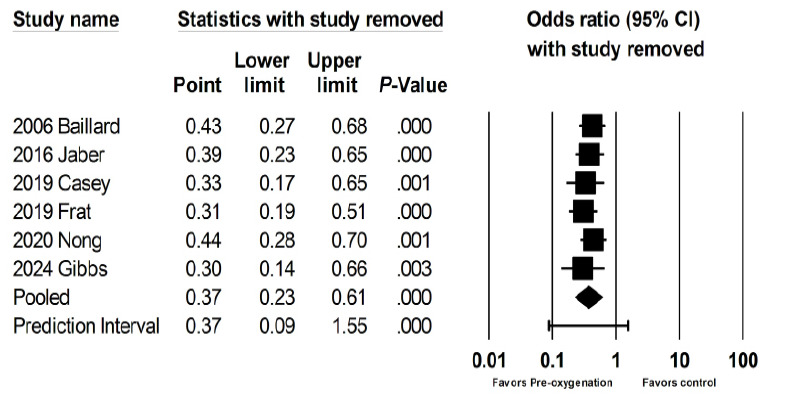
Sensitivity analysis for the association of preoxygenation techniques with rates of peri-intubation hypoxemia.

**Figure 3f:**
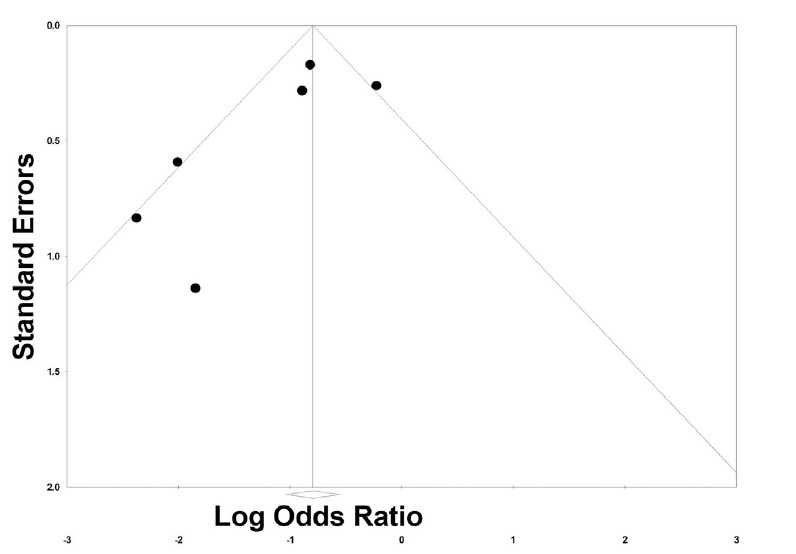
Publication bias funnel plot of studies investigating preoxygenation techniques on hypoxemia.

**Figure 4a:**
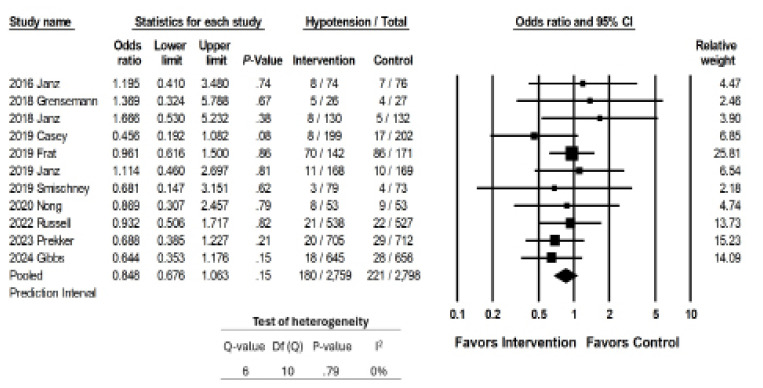
Association of interventions with rates of peri-intubation hypotension.

**Figure 4b:**
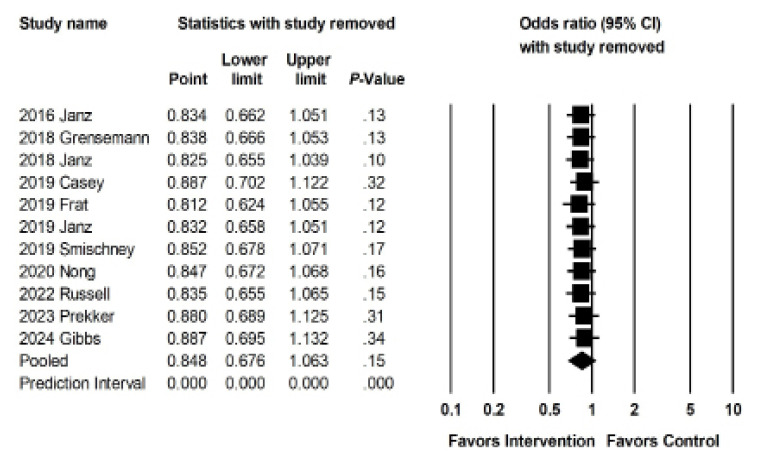
Sensitivity analysis for the association of interventions with rates of peri-intubation hypotension.

**Figure 4c:**
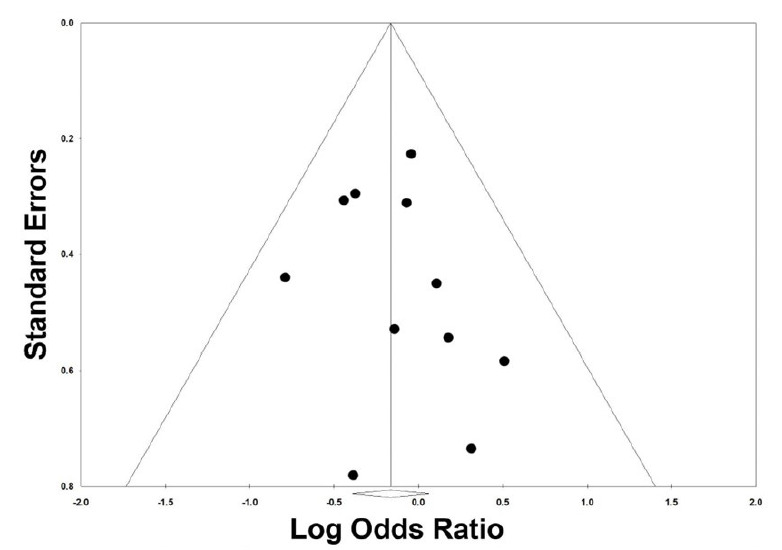
Publication bias funnel plot of studies investigating interventions on hypotension.

**Figure 5a:**
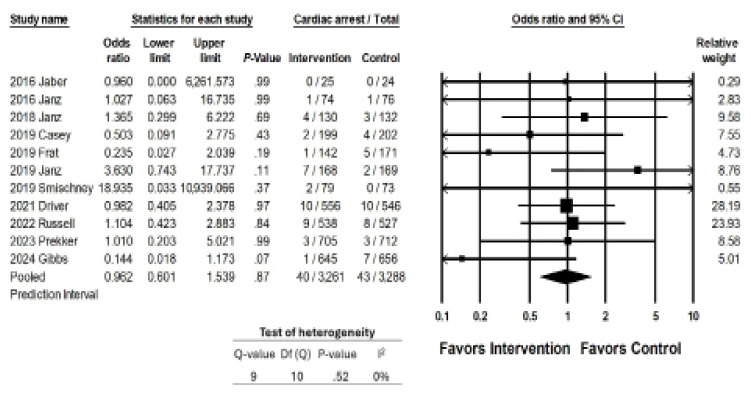
Association of interventions with rates of cardiac arrest.

**Figure 5b:**
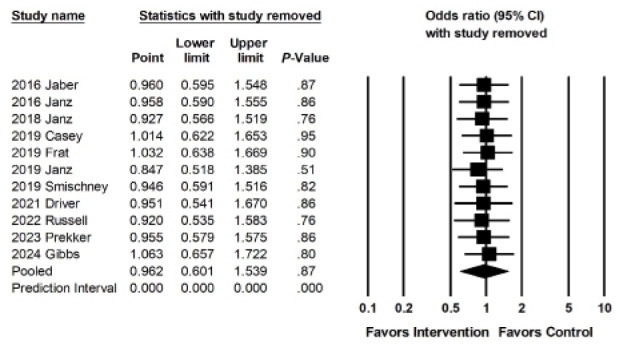
Sensitivity analysis for the association of interventions with rates of cardiac arrest.

**Table 1:** Moderator analysis for subgroup comparisons between studies, using categorical variables.

Moderator variables	Meta-analysis				Heterogeneity				Between group comparison P-value
	
	# of studies	OR	95% CI	P	Q-value	D(f)	P	I^2^	
Primary outcome 1: Hypoxemia									
WHO region									
AMR	11	0.85	0.65–1.12	.25	33	10	< .01	70%	
EUR	4	0.44	0.14–1.36	.15	9	3	.04	65%	.01
WPR	1	0.14	0.04–0.43	.001	N/a	N/a	N/a	N/a	
Intervention type									
Airway adjuncts^a^	5	1.07	0.78–1.5	.66	6	4	.17	38%	
Fluid bolus	2	1.02	0.76–1.4	.90	1	1	.38	0%	
NIV	4	0.35	0.17–0.72	< .01	12	3	.01	75%	< .01
Pre-oxygenation^b^	2	0.39	0.23–0.67	< .01	1	1	.52	0%	
VL	3	0.94	0.68–1.29	.69	1	2	.86	0%	
Sample size									
<100	3	0.29	0.05–1.8	.18	6	2	.04	70%	
<500	8	0.72	0.47–1.1	.13	21	7	< .01	66%	.45
>500	5	0.87	0.59–1.3	.49	21	4	< .01	81%	
Primary Outcome 2: Hypotension									
WHO Region									
AMR	8	0.79	0.60–1.05	.09	5	7	.62	0%	
EUR	2	0.99	0.65–1.5	.97	0.2	1	.65	0%	.69
WPR	1	0.87	0.31–2.5	.79	N/a	N/a	N/a	N/a	
Intervention type									
Airway adjuncts^a^	2	1.21	0.48–3.02	.41	0.8	1	.36	0%	
Fluid bolus	2	0.98	0.59–1.63	.96	0.1	1	.75	0%	
NIV	3	0.84	0.59–1.2	.31	1	2	.58	0%	.57
Pre-oxygenation^b^	1	0.46	0.19–1.08	.08	N/a	N/a	N/a	N/a	
VL	3	0.83	0.51–1.3	.45	1	2	.52	0%	
Sample size									
<100	1	1.4	0.3–5.7	.67	N/a	N/a	N/a	N/a	
<500	7	0.92	0.68–1.3	.60	4	6	.65	0%	.52
>500	3	0.74	0.53–1.05	.09	0.8	2	.67	0%	

a Includes patient positioning (ramped versus sniffing position), use of a gum-elastic bougie, and use of a standardized pre-intubation checklist.

b Includes non-invasive ventilation, bag-valve mask ventilation.

*AMR*, Regions of Americas; *DL*, direct laryngoscopy; *EUR*, Region of Europe; *HFNC*, high-flow nasal cannula; *ICU*, intensive care unit; *NIV*, non-invasive ventilation; *WHO*, World Health Organizations; *WPR*, Region of Western Pacific; *VL*, video laryngoscopy.

**Table 2:** Multivariate meta-regression evaluating the association between select continuous variables and peri-intubation major adverse events.

Variables	Corrected Coefficient (95% CI)	P	R^2^	I^2^
Outcome: Hypoxemia				
Age	0.03 (−0.03 to 0.09)	.36	0.15	65%
% Female	−0.26 (−4.7 to 4.2)	.91		
**BMI**	**0.35 (0.09 to 0.61)**	**.01**		
SBP prior to intubation	−0.003 (−0.04 to 0.03)	.89	0	80%
% of vasopressor prior to intubation	3.4 (−3.9 to 10.6)	.36	0	75%
% receiving low oxygen support	2.7 (−1.7 to 7.2)	.23	0.39	62%
% receiving NIV	0.41 (−3.8 to 4.6)	.85		
% receiving HFNC	−0.3 (−10 to 9.7)	.95	0.54	52%
**% of intubation for respiratory failure**	−**4.9 (**−**9.1 to** −**0.77)**	**.02**		
% of intubation for airway protection	0.49 (−3.3 to 4.4)	.81	0	52%
% of intubation for hemodynamic instability	−8.5 (−23.1 to 6.2)	.25	0.27	34%
% of intubation for other reasons	3.8 (−5.6 to 13.2)	.43	0.78	66%
Outcome: Hypotension				
Age	0.01 (−0.04 to 0.07)	.61		
% Female	−2.9 (−7.1 to 1.3)	.18	0	0%
BMI	0.04 (−.17 to 0.24)	.74		
SBP prior to intubation	−0.02 (−0.06 tp 0.02)	.41	0	0%
% of vasopressor prior to intubation	−1.4 (−7.2 to 4.4)	.64	0	0%
% receiving low oxygen support	1.8 (−2.4 to 5.9)	.41	0	9%
% receiving NIV	−1.3 (−3.2 to 0.58)	.17	0	0%
% receiving HFNC	NA	NA	NA	NA
% of intubation for respiratory failure	0.4 (−2.6 to 3.4)	.81	0	0%
% of intubation for airway protection	−1.4 (−7.5 to 4.7)	.66	0	0%
% of intubation for hemodynamic instability				
% of intubation for other reasons	0.08 (−10.4 to 10.6)	.98	0	0%

Bolded values denote statistical significance.

*BMI*, body mass index; *SBP*, systolic blood pressure; *HFNC*, high-flow nasal cannula; *NIV*, non-invasive ventilation.

**Table 3:** Moderator analyses using categorical variables for subgroup analyses in studies evaluating preoxygenation techniques.

Moderator Variables	Meta-analysis				Heterogeneity				Between-group comparison P-value
	# of studies	Outcome	95% CI	P	Q-value	D(f)	P	I^2^	
Outcome: Hypoxemia									
WHO Region									
AMR	2	0.43	0.32–0.58	.001	0.045	1	.83	0%	
EUR	3	0.28	0.06–1.3	.11	7.5	2	.02	73%	0.14
WPR	1	0.14	0.04–0.43	.001	NA	NA	NA	NA	
Clinical settings									
ICU	5	0.3	0.14–0.66	.003	13	4	.009	70%	0.38
Mixed settings	1	0.44	0.42–0.62	.001	NA	NA	NA	NA	
Sample size									
<100	2	0.11	0.03–0.42	.001	0.14	1	.71	0%	
<500	3	0.41	0.18–0.52	.032	9	2	.01	76%	0.14
>500	1	0.44	0.32–0.62	.001	NA	NA	NA	NA	
Intervention Type									
NIV	4	0.35	0.17–0.72	.004	12	3	.006	76%	0.81
Other pre-oxygenation	2	0.39	0.23–0.67	.001	0.6	1	.42	0%	

*AMR*, Regions of Americas; *EUR*, Region of Europe; *NIV*, non-invasive ventilation; *WHO*, World Health Organizations; *WPR*, Region of Western Pacific.

**Table 4:** Meta-regression using continuous variables and the outcome for odds of major adverse events among patients undergoing pre-oxygenation.

Variables	Number of studies	Corr. Coeff. (95% CI)	P
Outcome: Hypoxemia			
Age	5	0.02 (−0.23 to 0.28)	.84
Percentage of female		9.4 (−22.3 to 41.1)	.56
Body Mass Index	5	0.23 (−0.01 to 0.47)	.07
Percentage receiving low oxygen support	5	−2.4 (−6.8 to 1.9)	.28
Percentage receiving NIV	4	−1.2 (−3.5 to 1.2)	.32
Percentage receiving HFNC	NA	NA	NA
Percentage of intubation for respiratory failure	NA	NA	NA
Percentage of intubation for airway protection	NA	NA	NA

*Corr. Coeff*., Correlation Coefficient; *HFNC*, high-flow nasal cannula; *NIV*, non-invasive mechanical ventilation; *NA*, not enough studies reporting the variables for the analysis.
