# Supplementary material for: Impact of Interventions on Peri-Intubation Hypoxemia and Hypotension in Critically Ill Patients: Systematic Review and Meta-Analysis
Source: West J Emerg Med. 2025 Sep 27;26(5):1380–91. doi: 10.5811/westjem.41210 (PMC12591638; doi:10.5811/westjem.41210)
Supplement: Supplementary file 1 [file wjem-26-1380-s001.docx]

**Table 1.** Characteristics, quality assessments, and patient composition of RCTs included in the meta-analysis to evaluate the effect of interventions on the rate of peri-intubation MAEs.

|  |  |  |  |  |  |  | **Control** | | | | | | **Intervention** | | | | | |
| --- | --- | --- | --- | --- | --- | --- | --- | --- | --- | --- | --- | --- | --- | --- | --- | --- | --- | --- |
| **First author, year, country** | **Design** | **Location of intubation** | **Risk of Bias^a^** | **Control** | **Intervention** | **Primary outcome** | **N** | **Age, mean (SD)^b^** | **Female N (%)** | **BMI^c^** | **1^o^ outcome of interest** | **2o outcomes or complications relevant to this review** | **N** | **Age, mean (SD)^b^** | **Female N (%)** | **BMI^c^** | **1^o^ outcome of interest** | **2o outcomes or complications relevant to this review** |
| Baillard 2006 France | RCT | ICU | Low | NRB | NIV | Lowest SpO2 | 26 | 60 (15) | 7 (27%) | N/a | 81% (15 ) | SpO2 < 80% in 46% | 27 | 64 (11) | 10 (37%) | N/a | 93% (8) | SpO2 <80% in 7% |
| Casey 2019 USA | RCT | ICU | Low | NC | BVM | Lowest SaO2 | 202 | 59 (5.8) | 94 (47%) | 27.6 (23.4-34.2) | 93% | SpO2 < 80% in 23% | 199 | 57.5 (6.3) | 81 (41%) | 27.1 (22.7-32.3 | 96% | SpO2 <80% in 11% |
| Driver 2018 USA | RCT | ED | Low | ETT with stylet | Bougie | First Pass Success with Difficult Airway | 376 | 46 (18) | 121 (32%) | 28 (7) | 82% (150/182) | First pass success all-comers in 87% | 381 | 46 (18) | 109 (29%) | 28 (7) | 96% (191/198) | First pass success all-comers in 98% |
| Driver 2021 USA | RCT | ED, ICU | Low | ETT with stylet | Bougie | First Pass Success | 546 | 56.8 (6.6) | 229 (42%) | 26.6 (22.7-31.3) | 83% (453/546) | SpO2 < 80% in 11% | 556 | 56.8 (7.9) | (40%) 223 | 26.1 (22.7-31.3) | 80% (447/556) | SpO2 < 80% in 9% |
| Frat 2019 France | RCT | ICU | Low | HFNC | NIV | SpO2 < 80% during intubation | 171 | 64 (13) | 41 (29%) | 27 (6) | 27% (47/171) | None | 142 | 64 (14) | 60 (35%) | 27 (7) | 23% (33/142) | None |
| Gibbs 2024 USA | RCT | ED, ICU | Low | Oxygen mask | NIV | SpO2 <85% during intubation | 656 | 59.8 (6.6) | 260 (40%) | 26.6 (22.5-32.4) | 18.5% (118/637) | Lowest SpO2 after intubation 97% | 645 | 60 (6.9) | 40% (255) | 27.6 (23.3-32.9) | 9.1% (57/624) | Lowest SpO2 after intubation 99% |
| Grensemann 2018 Germany | RCT | ICU | Some concerns | DL | VL | First Pass Success | 27 | 57 (14) | 13 (48%) | N/a | 93% (25/27) | None | 26 | 63 (15) | 11 (42%) | N/a | 96% (25/26) | None |
| Jaber 2016 France | RCT | ICU | Low | NIV | NIV + HFNC | Lowest SpO2 | 24 | 60.8 (3.5) | 4 (17%) | 23 (20-28) | 96% (92-99) | None | 25 | 62.8 (3.2) | 7 (28%) | 24 (20-29) | 100 | None |
| Janz 2016 USA | RCT | ICU | Low | DL | VL | First Pass Success | 76 | 59.5 (4.6) | 32 (42%) | 28.8 (23.1-33.3) | 65.8% (50/76) | In hospital mortality 42% | 74 | 58.8 (5.5) | 27 (36%) | 28.5 (23.4-32.7) | 68.9% (51/74) | In hospital mortality 41% |
| Janz 2018 USA | RCT | ICU | Low | Usual care | Checklist | Lowest SpO2 and lowest SBP | 132 | 57.8 (6.1) | 54 (41%) | 27.4 (23.8-33.4) | 93% (84-100); 108 (90-132) | None | 130 | 55.5 (4.6) | 43 (33%) | 27.4 (23.8-33.4) | 92% (79-98); 112 (94-133) | None |
| Janz 2019 USA | RCT | ED, ICU | Low | No bolus | IVF bolus | Cardiovascular collapse | 169 | 57.5 (6.3) | 73 (43%) | 26 (22-32) | 18% (31/169) | None | 168 | 59.8 (6.6) | 81 (48%) | 27 (23-32) | 20% (33/168) | None |
| Nong 2020 China | RCT | ICU | Low | BVM | NIV | Lowest SpO2 and percent with SpO2 <80% | 53 | 66 (13) | 16 (30%) | N/a | 83% (74-91); 38% (20/53) | Mean intubation time 2.2 minutes (2.5) | 53 | 63 (12) | 15 (28%) | N/a | 95% (87-100); 7% (4/53) | Mean intubation time 1.6 minutes (1.3) |
| Prekker 2023 USA | RCT | ED, ICU | Low | DL | VL | First Pass Success | 712 | 55 (39-67) | 258 (36%) | 26.5 (23-31.6) | 71% (504/712) | Severe complications during intubation^d^ in 20.9% | 705 | 54 (36-66_ | 240 (34%) | 26.3 (22.7-31.4) | 85% (600/705) | Severe complications during intubation^d^ in 21.4% |
| Russell 2022 USA | RCT | ICU | Low | No bolus | IVF bolus | Cardiovascular collapse | 527 | 61.5 (5.8) | 228 (43%) | 27.7 (23.5-32.7) | 19% (96/527) | Death prior to day 28 in 42.3% | 538 | 60.8 (5.5) | 220 (41%) | 27.7 (23.5 - 32.7) | 21% (113/538) | Death prior to day 28 in 40.5% |
| Semler 2017 USA | RCT | ICU | Low | Sniffing position | Ramped position | Lowest SaO2 | 130 | 56 (45-64) | 51 (39%) | 27.3 (24-32.6) | 92% (79-98) | Grade III/IV view (12%); difficult intubation (5%); first attempt (85%) | 130 | 56 (47-65) | 51 (39%) | 26.7 (23.9-33.3) | 93% (84-99) | Grade III/IV view (25%); difficult intubation (12%); first attempt (76%) |
| Smischney 2019 USA | RCT | ICU | Low | Reduced dose etomidate | Ketamine + Propofol | Change in MAP from baseline in 5 mins | 73 | 60 (18) | 35 (48%) | 27.5 (6.5) | 81.7 ±17.2 mmHg vs. baseline of 82.8 ±17.9 mm Hg | None | 79 | 62 (17) | 31 (39%) | 28 (6.3) | 77.6 ±15.4 mmHg vs. baseline of 80.9 ±13.3 mmHg | None |

^a^ Assessed using the Cochrane risk-of-bias 2 tool.

^b^ Studies that used median and interquartile values were converted to mean and standard deviation using VassarStats.^32^ The following studies were converted: Casey 2019,^13^ Driver 2021,^17^ Gibbs 2024,^18^ Jaber 2016,^22^ Janz 2016,^28^ Janz 2018,^16^ Janz 2019,^14^ Russell 2022,^26^ and Semler 2017.^15^

^c^ Data is presented as medians (interquartile range) or mean (SD)*.*

^d^ Described by authors as composite of SpO2 < 80%, SBP<65, new or increased vasopressors, cardiac arrest, death.

*Abs,* absolute; *BVM,* bag-valve-mask; *diff,* difference; *DL,* direct laryngoscopy; *ETT,* endotracheal tube; *IVF,* intravenous fluid; *HFNC,* high-flow nasal cannula; *NC,* nasal cannula; *NIV,* non-invasive ventilation; *NRB,* nonrebreather mask; *RCT,* randomized controlled trial; *RR,* relative risk; *SaO2,* arterial oxygen saturation; *SBP,* systolic blood pressure; *SpO2,* peripheral capillary oxygen saturation; *VL,* video laryngoscopy.
